# Agile Blocker and Clock Jitter Tolerant Low-Power Frequency Selective Receiver with Energy Harvesting Capability

**DOI:** 10.1038/s41598-017-10023-8

**Published:** 2017-08-29

**Authors:** Abul Hasan, Mohamed Helaoui, Fadhel M. Ghannouchi

**Affiliations:** iRadio Laboratory, Department of Electrical and Computer Engineering, Schulich School of Engineering, University of Calgary, T2N 1N4 Calgary, Alberta Canada

## Abstract

In this article, a novel tunable, blocker and clock jitter tolerant, low power, quadrature phase shift frequency selective (QPS-FS) receiver with energy harvesting capability is proposed. The receiver’s design embraces and integrates (i) the baseband to radio frequency (RF) impedance translation concept to improve selectivity over that of conventional homodyne receiver topologies and (ii) broadband quadrature phase shift circuitry in the RF path to remove an active multi-phase clock generation circuit in passive mixer (PM) receivers. The use of a single local oscillator clock signal with a passive clock division network improves the receiver’s robustness against clock jitter and reduces the source clock frequency by a factor of N, compared to PM receivers using N switches (N≥4). As a consequence, the frequency coverage of the QPS-FS receiver is improved by a factor of N, given a clock source of maximum frequency; and, the power consumption of the whole receiver system can eventually be reduced. The tunable QPS-FS receiver separates the wanted RF band signal from the unwanted blockers/interferers. The desired RF signal is frequency down-converted to baseband, while the undesired blocker/interferer signals are reflected by the receiver, collected and could be energy recycled using an auxiliary energy harvesting device.

## Introduction

The increasing demand for wireless connectivity and the overcrowding of frequency spectrum with tightly packed signals have resulted in growing demand for blocker and interferer tolerant frequency selective radio receivers (Rx) with tuning capability over a wide frequency range with increasingly reduced power consumption. There is also a growing concern for energy in the future development of wireless devices and networks^1–3^. Although, the proliferation of wireless nodes radiating radio frequency (RF) power poses design challenges for Rx, it also opens up a new window of opportunity for energy harvesting/scavenging, where new design and implementation techniques can increase the battery life of wireless devices or the devices can be made to operate solely from the power harvested from many other sources of energy, including ambient RF power^1–5^.

There are a variety of possible implementations of frequency selective and tunable Rx architectures in the published literature, including the conventional quadrature down-conversion receiver (zero or low intermediate frequency (IF) or superheterodyne) with tunable RF bandpass filters and components^6–10^, the direct sampling receiver^11^, the subsampling receiver^12, 13^, the multi-port receiver^14–16^, and the passive mixer (PM) based receiver^17–20^. The PM architecture is a zero (or low) IF design that has caught the attention of researchers by having the potential of (a) concurrent RF filtering and frequency down-conversion, (b) robustness to 1/*f* noise for a zero-IF configuration, (c) linear operation, compared to other mixer-based receiver architectures, (d) highly selective filtering over a very wide RF frequency band with fixed/tunable bandwidth, (e) simultaneous and superior interferer/blocker filtering along with in-phase/quadrature phase (*I*/*Q*) demodulation, and (f) superior interferer and blocker rejection performance.

Intensive research studies have also been carried out in the areas of ambient RF energy harvesting/scavenging^21–24^. Rectifier-based approaches using diodes and transistors^21–23^ and time-reversal duality-based approaches for power amplifiers/rectifiers^24, 25^ are some of the methods through which efficient RF energy harvesting techniques have been implemented. Wireless energy harvesting has a promising application in the emerging field of Internet of Things (IoT) where energy aware devices are highly desirable in order to have longer autonomy without the need for recharging^26^. Furthermore, the reconfigurable selectivity of the proposed topology makes it a good candidate for software defined radio and multi-standard/multi-band wireless applications.

**Figure 1:**
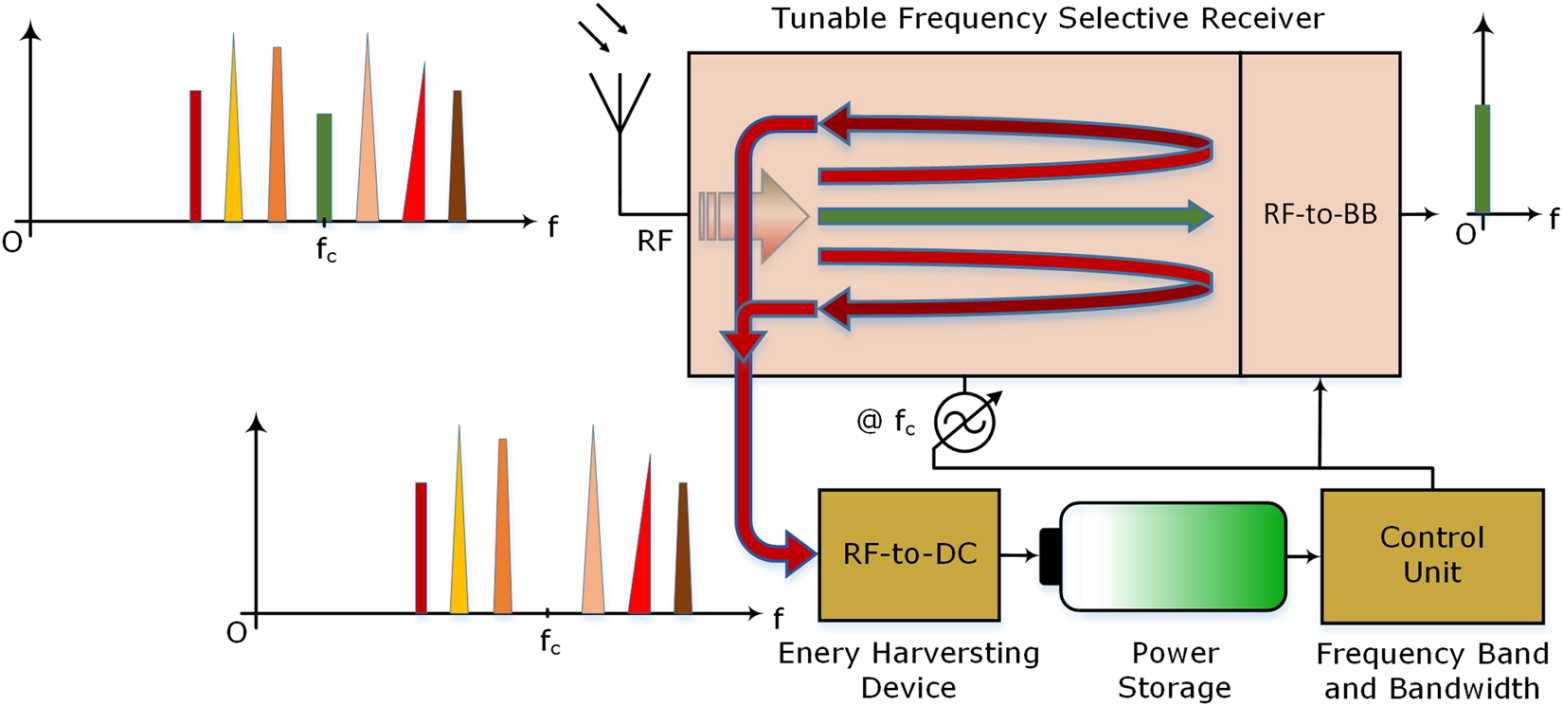
Tunable blocker tolerant frequency selective energy harvesting enabled receiver. A simplified block diagram of a tunable frequency selective receiver with energy harvesting capability is illustrated. The center frequency and bandwidth of operation for the receiver are tunable: only the RF band signal present at the frequency of operation in a set bandwidth is frequency down-converted by the receiver, while all the other unwanted blockers and interferer signals are separated from the desired band signal and used in an energy harvesting device (RF-to-DC) for converting ambient RF radiated power to direct current power for storage and usage. The receiver operating frequency, down-conversion bandwidth and other parameters are set by the control unit.

The main focus of this article is the proposal of a new architecture for Rx design that is low power and tolerant of many system and environmental non-idealities with energy harvesting capability by energy recycling the unwanted interferers. In the proposed design, the wanted RF band signal is efficiently frequency down-converted to baseband (BB), while the unwanted ambient RF signals are collected and can be used in an energy harvesting system for power generation and storage. To the best of the authors’ knowledge, there has been no other such Rx topology that is low power, blocker and clock jitter tolerant, while also isolating and removing unwanted blockers and interferers, from the desired band RF signal, which can be used further in an energy harvesting system. The desired band RF signal is frequency down-converted to baseband in the receiver; and, the blockers and interferer signals are supplied to an energy harvesting device, where they would be converted to direct current (DC) power for storage and reuse. The concept of the proposed radio receiver topology is shown in Fig. 1.

## Method

### Impedance Translation, Frequency Down-conversion and Passive Mixing

**Figure 2:**
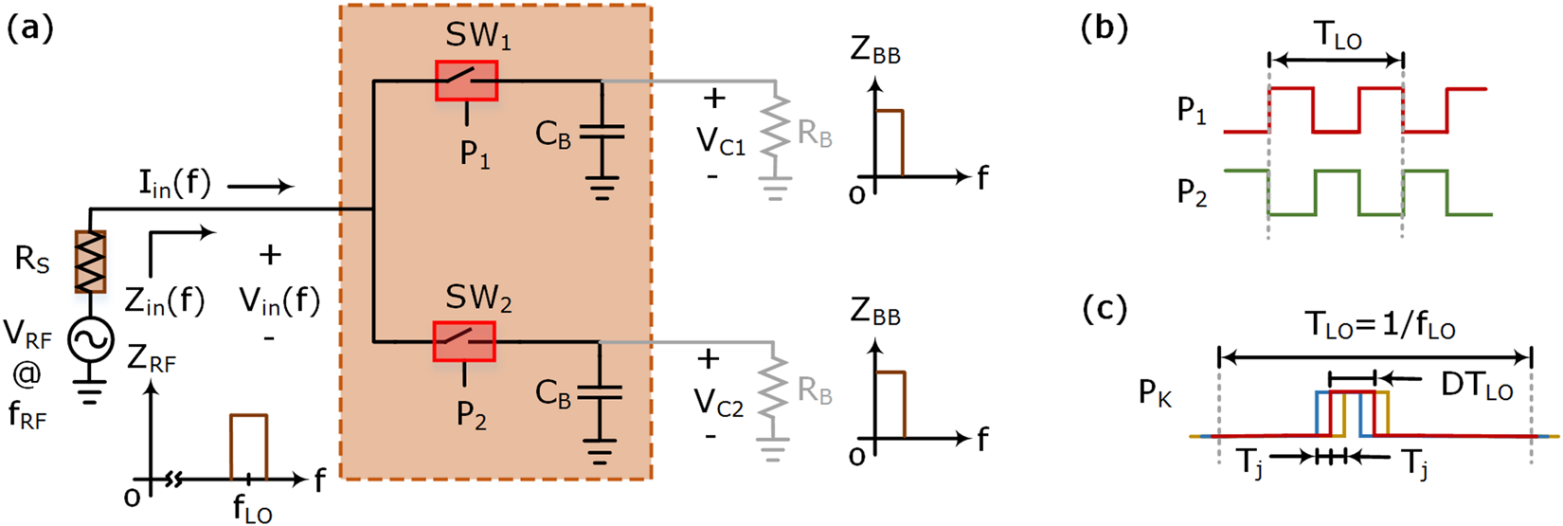
Impedance translation circuit. (**a**) Simplified block diagram of a voltage mode impedance translation switching network using two switches and baseband impedances (*C*_*B*_ and *R*_*B*_). The switches are operated on and off periodically by **(b)** two non-overlapping pulse waveforms each of duty cycle *D*, where (**c**) a switching pulse waveform is associated with its timing jitter (*T*_*j*_) characterized as the fluctuation of reference edges of a clock signal with respect to their ideal positions in time.

Impedance translation is usually defined as the translation of impedance (or transfer function) of a network present at one frequency to another, with the help of some periodically driven time variant system^27^. One of the simplest circuits that can perform a tunable impedance translation is a voltage mode impedance translation circuit, which is comprised of a parallel combination of two switches and driven periodically by two non-overlapping pulse waveforms of duty cycle *D* (usually 0.5), with one common RF terminal, and the other switch terminals connected to their respective baseband impedances, capacitors of value *C*_*B*_ in parallel with resistor *R*_*B*_, as shown in Fig. 2. When a broadband ideal voltage signal source with output impedance *R*_*S*_ (antenna with its input impedance of 50 Ω for receiver applications) is connected to this network, the voltage signal source sees an impedance translation of low-pass baseband impedance (*C*_*B*_||*R*_*B*_ in series with the voltage source impedance and the switch on-resistance) to a new bandpass input impedance of the network ${Z}_{in}(f)={V}_{in}(f)/{I}_{in}(f)$ at RF^17–19^, as shown in Fig. 2a. It is assumed that the switches are identical with finite low on-resistance (*R*_*SW*_), very high off-resistance, and the clock duty cycle (*D*) is such that effectively only one of the two switches is on at any given time.

The two-path impedance translation circuit, shown in Fig. 2a, exhibits two different input impedance values for the input RF signals in two different RF bands. The passband input impedance, defined as the input impedance of the impedance translation circuit at *f*_*RF*_ = *f*_*LO*_ is approximated by Eq. (1)^28^, where *f*_*LO*_ is the switching frequency of the clock pulse waveforms. The stopband input impedance, which is defined as the input impedance of the network outside the passband of the impedance translation circuit, is approximated by Eq. (2)^28^.

1 $${Z}_{in,passband}(f)=\frac{{R}_{S}+{R}_{SW}}{2D[1-{(\frac{\sin \pi D}{\pi D})}^{2}]}-{R}_{S}$$

2 $${Z}_{in,stopband}(f)=\frac{{R}_{S}+{R}_{SW}}{2D}-{R}_{S}$$

In order to perform concurrent impedance translation from baseband to RF frequency (*f*_*RF*_ = *f*_*LO*_) and *I*/*Q* demodulation of a bandlimited RF signal present at *f*_*RF*_ = *f*_*LO*_ to baseband, a network of minimum four switches is required, where these four switches are driven periodically at speed *f*_*LO*_ using four non-overlapping time delayed pulse waveforms shifted progressively in time by *T*_*LO*_/4. This approach of impedance translation and I/Q demodulation is used in the conventional passive mixer based receiver systems^17–20, 29–34^. The four non-overlapping time delayed pulse waveforms are generated using an active multiphase clock generator circuit that takes a single higher speed clock signal at frequency 4*f*_*LO*_ and converts it into four non-overlapping clock pulse waveforms of reduced speeds (*f*_*LO*_) and duty cycles. The output voltages of the baseband capacitors are phase-shifted and combined to allow demodulation of a desired RF band signal and generate the demodulated I and Q components of the baseband signal^18, 34^. In passive mixers, time jitters associated with clocks result in pulse overlaps, which contribute to conversion loss and also degraded output signal quality.

### Proposed Receiver System

Electronic information radiated from modern wireless transmitters is modeled by Eq. (3), where *I*(*t*) and *Q*(*t*) are the baseband I and Q components, respectively, and *f*_*RF*_ is the carrier frequency, of modulated transmitted RF signal *r*(*t*).

3 $$r(t)=\mathrm{Re}\{[I(t)+jQ(t)]{e}^{j2\pi {f}_{RF}t}\}=I(t)\cos (2\pi {f}_{RF}t)-Q(t)\sin (2\pi {f}_{RF}t)$$

A fundamental function of the Rx is to faithfully recover the information contained in the *I*(*t*) and *Q*(*t*) signals from the received version of signal *r*(*t*) at frequency *f*_*RF*_.

**Figure 3:**
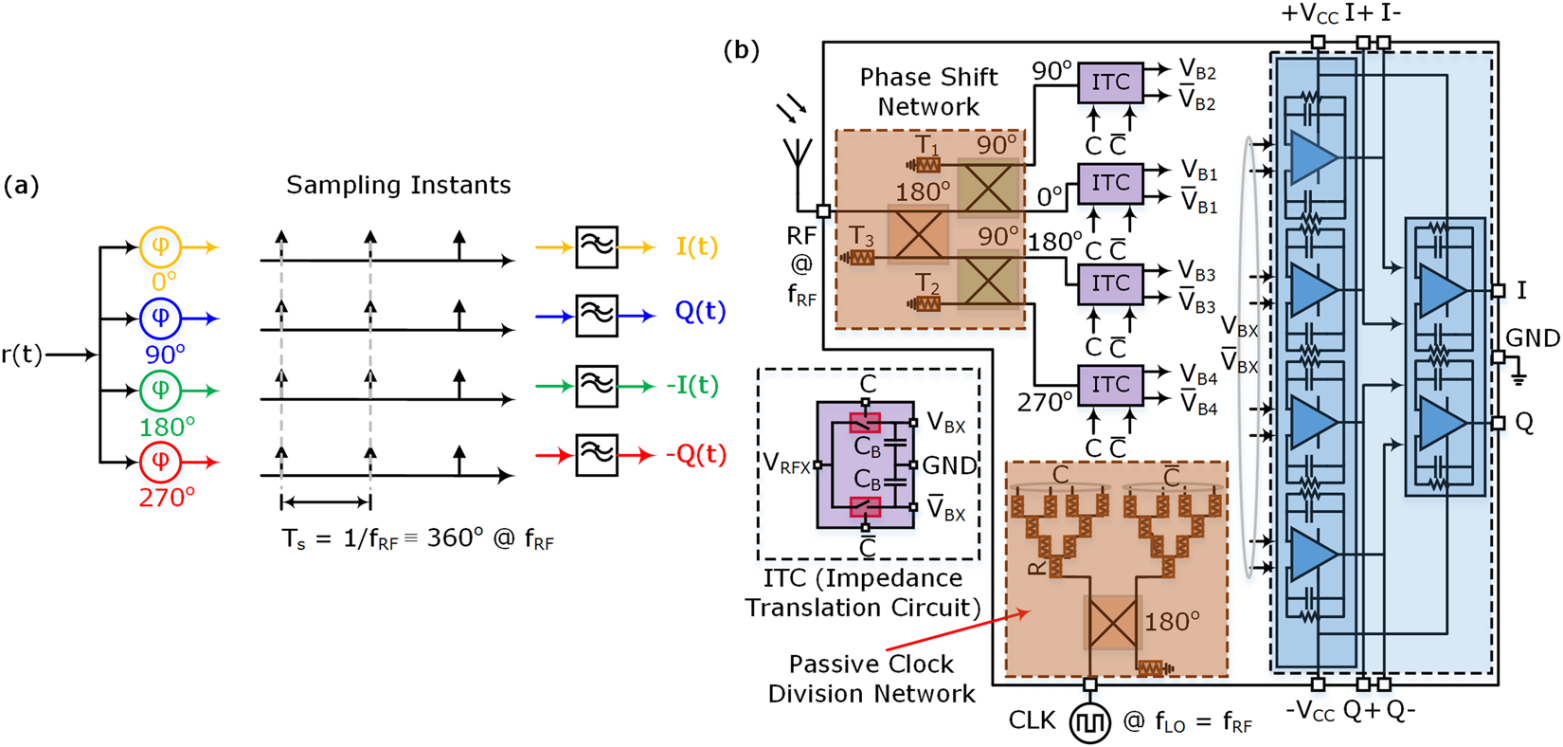
Sampling scheme and proposed QPS-FS receiver. **(a)** Equivalent sampling scheme used in the proposed QPS-FS receiver, where a single sampling clock pulse waveform is used to sample four different phase-shifted versions of the received RF signal. **(b)** Simplified block diagram of the proposed receiver system using the new phase shift and sampling scheme, and an impedance translation circuit (ITC) using two switches. The RF and local oscillator (LO) paths are divided, and the resultant baseband voltage signals are combined to obtain I and Q components of a received RF signal.

We propose a new approach for concurrent I/Q demodulation and impedance translation that provides some advantages over the conventional passive mixers and homodyne receivers. The proposed approach generates four copies of phase-shifted versions of the received signal *r*(*t*) at carrier frequency *f*_*RF*_ and utilizes a single clock pulse waveform of frequency *f*_*RF*_ for sampling all the four versions of the RF signal as shown in Fig. 3a.

If ${\phi }_{i}(i=1,2,\mathrm{...},4;{\phi }_{i}=(i-1)\times {90}^{{\rm{o}}})$ is the phase shift introduced by the ${i}^{th}$ path of the received *r*(*t*) (assuming no noise/interference and distortion), the resultant ${i}^{th}$ path RF signal is provided by Eq. (4).

4 $${r}_{i}(t)={\rm{R}}{\rm{e}}\{[I(t)+jQ(t)]{e}^{j(2\pi {f}_{RF}t-{\phi }_{i})}\}=I(t)\cos (2\pi {f}_{RF}t-{\phi }_{i})-Q(t)\sin (2\pi {f}_{RF}t-{\phi }_{i})$$

If the received signal is sampled at a sampling speed of *f*_*RF*_, the sampled version of the RF signal obtained from the ${i}^{th}$ path is given by Eq. (5). The bandwidths $(B)$ of signals $I(t)$ and $Q(t)$ (and hence *r*(*t*)) are assumed to be very small compared to ${f}_{RF}(B\ll {f}_{RF})$, so that the effective sampling of signals $I(t)$, $-Q(t)$, $-I(t)$ and $Q(t)$ at a sampling rate of *f*_*RF*_ do not introduce any significant problems with aliasing or noise $(B\ll {f}_{RF})$.

5 $${r}_{i}[k]=I[k]\cos \{2\pi {f}_{RF}(\frac{k}{{f}_{RF}})-{\phi }_{i}\}-Q[k]\sin \{2\pi {f}_{RF}(\frac{k}{{f}_{RF}})-{\phi }_{i}\}=\{\begin{array}{c}I[k];i=1\\ Q[k];i=2\\ -I[k];i=3\\ -Q[k];i=4\end{array}$$

Utilizing this sampling approach and the impedance translation approach using two switches described above, we propose a quadrature phase shift frequency selective (QPS-FS) receiver that alleviates some of the problems of conventional homodyne and passive mixer receivers. Moreover, the proposed receiver can be fitted with an auxiliary RF-to-DC rectifier to make it suitable for concurrent energy harvesting from ambient RF radiation.

In the proposed QPS-FS receiver, the four phase-shifted versions of the RF signal are generated using a phase shift network comprising of one 180° hybrid coupler and two 90° quadrature hybrid couplers. The four output ports of this phase shift network are terminated with impedance translation circuits (ITC) using two switches and two capacitors. A block diagram of the proposed QPS-FS receiver is shown in Fig. 3b.

When the isolation port of a hybrid coupler is properly terminated (matched to its characteristic impedance), the input port impedance of the coupler is matched to the characteristic impedance of the system, given that the two output ports of the coupler are terminated with loads of equal impedance values, not necessarily the characteristic impedance of the system. In this case of identical, but non-characteristic, impedance termination of the output ports of an isolated coupler, the input port remains matched to the characteristic impedance; and, the phase relation between the two output ports is maintained throughout the frequency band of operation, although the output power transfers to the terminating loads may not be optimal. These properties of the couplers are exploited and used in the phase shift and clock division networks of the proposed receiver architecture.

The QPS-FS receiver utilizes the voltage mode impedance translation circuit shown in Fig. 2a. The impedance mismatch between the phase shift network output ports and the ITC inputs can be assessed as an advantage for voltage conversion gain, as only the input voltage is frequency down-converted and stored on the output capacitors. The current and power transfer become irrelevant parameters in this voltage mode mixing and impedance translation.

The output voltages of the capacitors (${V}_{BX}/{\overline{V}}_{BX}$) are demodulated signals $I(t)$, $-Q(t)$, $-I(t)$ or $Q(t)$, depending on the RF phase shift path and the switching transistor where the output signal is taken. The eight output signals from the ITCs in Fig. 3b are further filtered and processed analogically (differential amplifiers) or digitally (high impedance analog-to-digital-converter (ADC) followed by a digital-signal-processor (DSP)), and the frequency down-converted and demodulated baseband signals $I(t)$ (or $I[n]$) and $Q(t)$ (or $Q[n]$) are obtained.

As shown in Fig. 3b, the antenna is connected directly to the phase shift network, which allows for any interferers or blockers falling within the intended frequency band of coverage of the receiver to undergo similar amplitude and phase shifts by the phase shift network as originally planned for the desired RF band signal. When this phase-shifted combination of the desired RF signal and the undesired interferes reach the junction of the phase shift network output and the ITC input, the desired RF band signal is frequency down-converted into the output capacitor voltages as baseband signals, while the interferers are reflected back and absorbed into termination resistors ${T}_{1}$, ${T}_{2}$, and ${T}_{3}$, as shown in Fig. 3b. The interferer signals reaching ${T}_{1}$, ${T}_{2}$ and ${T}_{3}$ may be energy recycled by combining all the interferers and blockers and suppling them to a wideband energy harvesting system for DC power generation and storage.

### Measurement Setup

**Figure 4:**
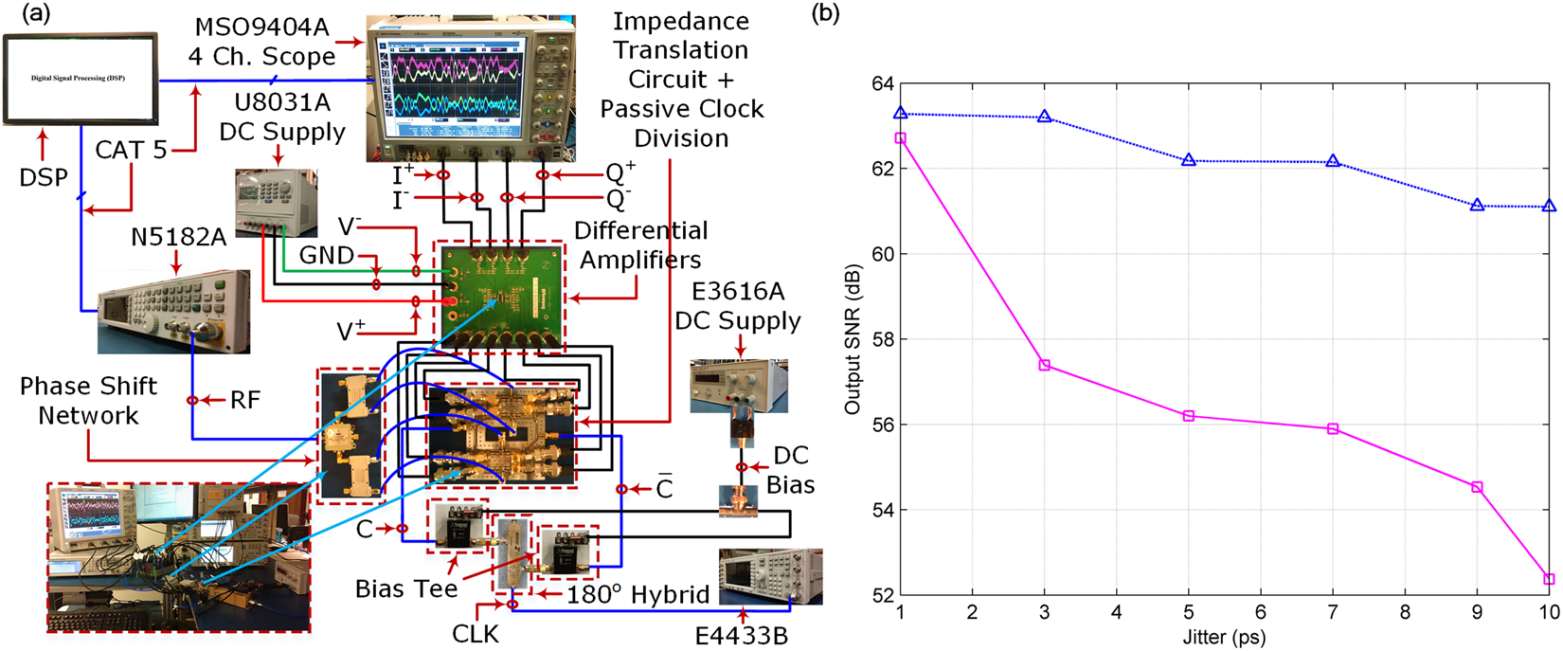
Measurement setup and simulation result. (**a**) Basic measurement setup for the proposed QPS-FS receiver. The RF phase shift and clock division networks were implemented using off-the-shelf passive RF components. The ITCs and a part of the clock division network were designed on a single PCB. (**b**) Simulated output signal-to-noise ratio (SNR) at 1.0 GHz operating frequency with clock jitter values $({\sigma }_{j})$ for the conventional differential passive mixer receiver () and proposed QPS-FS receiver (). The better output SNR for the proposed receiver confirms its jitter tolerant behavior.

A real test setup was developed to empirically validate the proposed QPS-FS receiver. Figure 4a shows a basic test setup that was used to implement and verify the workings of the QPS-FS receiver concept. Appropriate modifications in this basic test setup were made to measure and characterize different behaviors (e.g., intercept points, blocker behavior) of the proposed receiver system. Measurement specific modifications to this basic test setup are described in the next section. ITCs were designed using enhanced mode pHEMT (pseudomorphic high-electron-mobility transistor) GaAs-FET (gallium arsenide – field-effective transistor) ATF55143 transistors from Avago Technologies, Inc. Multilayer ceramic RF capacitors from American Technical Ceramics (ATC), Inc. were used to hold the frequency down-converted baseband voltage signals.

The outputs from the ideal clock voltage source can be directly connected to the high input impedance gate terminals of the switching transistors. Due to the unavailability of a square wave clock voltage generator, a continuous wave (CW) clock source with 50 Ω output impedance was used in conjunction with a passive clock division circuit, which was a 10 Ω resistive signal divider network in combination with a 180° hybrid coupler, as shown in Fig. 3b.

The ITCs and the passive clock division network were designed on the same printed circuit board (PCB) using a fiberglass reinforced epoxy (FR4) substrate with a thickness of 1.6 mm. Commercially available 180° and 90° hybrid couplers were used to implement the phase shift network and the passive clock divider hybrid, and a differential amplifier evaluation board was used to change the output mode from single-ended to differential.

In the implemented test setup, the clock source was a Keysight Technologies, Inc. E4433B CW RF signal generator, instead of an ideal square pulse wave generator used in the theoretical modeling and analysis. After passing the clock signal to a 180° degree hybrid to get two out-of-phase signals, the out-of-phase signals were passed through two bias tees, as shown in Fig. 4a, so that the resultant sinusoids traveling to the gates of the switching transistors provide switching behaviors for the transistors that were as close as possible to the ideal switch behaviors driven by square pulses.

For modulated signal based measurements, the baseband modulated signals were generated on a desktop computer in MATLAB^®^ and downloaded to a Keysight Technologies, Inc. N5182A vector signal generator to generate an RF modulated signal for the proposed receiver test system. The baseband output signals ($I+/-$ and $Q+/-$) from the receiver were captured using a four-channel oscilloscope (MSO9404A from Keysight Technolgies, Inc.) in the high input impedance mode. The captured baseband waveforms were processed and compared with the original transmitted baseband signals, and the receiver performance was evaluated in the DSP block shown in Fig. 4a. Other signal generators (E4433B/E4422B) from Keysight Technologies, Inc. were also used, and their outputs were combined using an off-the-shelf power combiner to generate two-tone CW RF signals and blockers for other tests and measurements.

## Results

Figure 4b shows the simulated performance of an ideal differential four-phase conventional PM receiver compared with the proposed QPS-FS receiver for 1.0 GHz band of operation. The baseband output signal was 1.0 MHz for the RF signal at 1.001 GHz and the clock switching frequency $({f}_{LO})$ was 1.0 GHz in the Advanced Design System (ADS) simulation software from Keysight Technologies, Inc. All the system elements used in the simulations were ideal components, except the clock sources. The only non-ideal elements in the simulation were the associated clock signals, which were characterized by their respective jitter values (${T}_{j}$) shown in Fig. 2c and their statistical distributions described according to Eq. (6). Each of the four clock signals involved in the conventional four-phase differential PM receiver had fixed 25% duty cycles with independent jitter values (${\sigma }_{j}$) according to Eq. (6). The clock signal involved in the QPS-FS receiver in Fig. 3b had a duty cycle of 50%, and its associated jitter value was also described according to Eq. (6). The simulation result shown in Fig. 4b confirms the clock jitter tolerant behavior of the proposed QPS-FS receiver compared to the conventional differential four-path passive mixer receiver.

6 $$f({T}_{j};0,{\sigma }_{j}^{2})=\frac{1}{{\sigma }_{j}\sqrt{2\pi }}{e}^{-\frac{1}{2}{(\frac{{T}_{j}}{{\sigma }_{j}})}^{2}}$$

**Figure 5:**
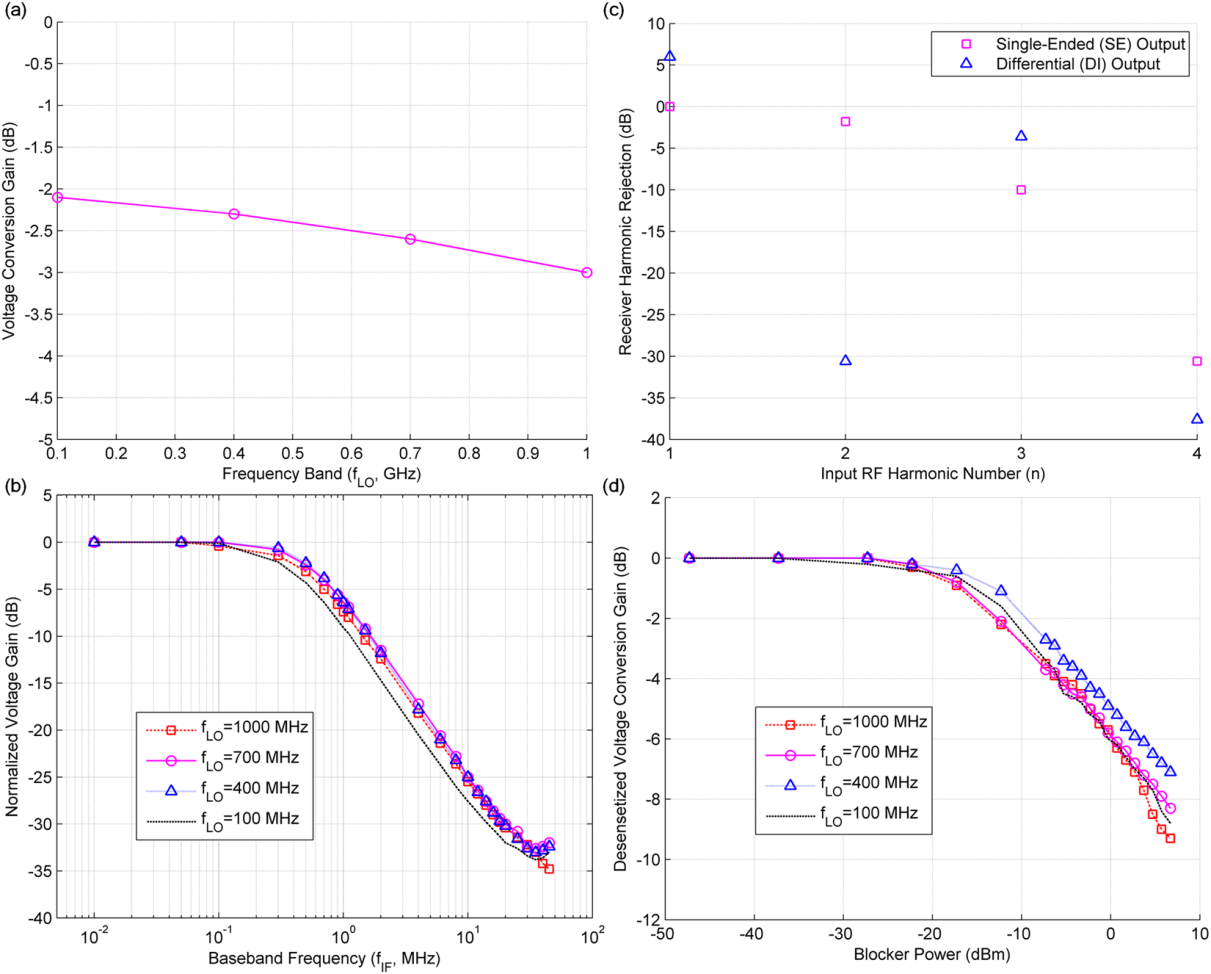
Measured performance of the proposed QPS-FS receiver. **(a)** Measured CW single-ended voltage conversion gain of the proposed receiver system. The frequency down-converted baseband frequency is fixed at ${f}_{IF}=$ 0.1 MHz for different frequency bands (${f}_{LO}$), and the CW RF signal is sent at frequency ${f}_{RF}={f}_{LO}+{f}_{IF}$. **(b)** Selectivity behavior of the QPS-FS receiver is illustrated by plotting the normalized voltage gain of the demodulated baseband output signal at frequency *f*_*IF*_ for different LO frequencies (*f*_*LO*_). The RF signal is sent at frequency *f*_*RF*_ = *f*_*LO*_ + *f*_*IF*_, where *f*_*LO*_ is fixed for the band of operation and *f*_*IF*_ is varied to characterize the receiver frequency selectivity behavior. **(c)** Harmonic rejection performance of the receiver; single-ended () and differential () baseband outputs relative to the single-ended baseband output of the desired RF band (*n* = 1) with harmonic *n* of the RF signal for 700 MHz band (*f*_*LO*_ = 700 MHz). The baseband output frequency is fixed at *f*_*IF*_ = 0.1 MHz, and the RF signal is sent at a frequency *f*_*RF*_ = *nf*_*LO*_ + *f*_*IF*_. **(d)** Receiver gain desensitization due to a CW blocker for different frequency bands of operation (*f*_*LO*_); the RF signal is sent at *f*_*RF*_ = *f*_*LO*_ + *f*_*IF*_, the CW blocker is at ${f}_{B}={f}_{LO}+$ 50 MHz, and the frequency down-converted desired baseband output signal is obtained at *f*_*IF*_ = 0.1 MHz.

CW measurements on the proposed receiver were performed to ascertain its frequency conversion and selectivity behavior compared to homodyne receivers. Unless specified, all the measurement results provided are for the single-ended output mode of the receiver. In order to obtain optimal conversion from RF to baseband voltages at the output capacitors, the clock amplitude and bias levels were adjusted. Figure 5a shows the measured single-ended voltage conversion gain of the QPS-FS receiver system when the RF signal level was fixed at −33 dBm, the baseband IF frequency (${f}_{IF}$) was fixed at 0.1 MHz, and the CW RF signal was present at frequency ${f}_{RF}={f}_{LO}+{f}_{IF}$ for different frequency bands of operation $({f}_{LO})$. The receiver input voltage value was derived from the input RF power level, assuming the receiver input was perfectly matched to the characteristic impedance of the phase shift network (50 Ω).

The frequency selectivity of the proposed receiver was measured for different RF bands of operation decided by ${f}_{LO}$, using a CW RF signal at frequency ${f}_{RF}={f}_{LO}+{f}_{IF}$, where ${f}_{LO}$ is fixed for the band of interest and *f*_*IF*_ is swept so that the frequency down-converted baseband signals fall at frequency *f*_*IF*_. The amplitude level of the baseband signal was recorded and plotted against *f*_*IF*_. The measured output frequency down-conversion exhibits bandpass filter behavior in the RF band and low-pass filter behavior in the baseband, as shown in Fig. 5b. In order to compare selectivity for different RF bands, the normalized baseband voltage gain (output baseband voltage at *f*_*IF*_ relative to that in the passband of the specific ${f}_{LO}$) was plotted against *f*_*IF*_, where ${f}_{LO}$ is fixed for the band of interest.

From the measured frequency selectivity characteristics of the proposed receiver system, the tunable RF bandpass selective behavior of the proposed QPS-FS receiver is confirmed. The bandwidth of the frequency down-conversion process is independent of the RF carrier frequency, depending only on the RF source impedance and the switch on-resistance values, baseband output capacitor value and the duty cycle of the clock pulse waveforms driving the switches.

Due to switching mixing, the RF signals present at ${f}_{RF}=n{f}_{LO}+{f}_{IF},n\in {\mathbb{N}}$ for any clock frequency (${f}_{LO}$) of the receiver in the QPS-FS receiver system are also frequency down-converted to *f*_*IF*_ for the single-ended output mode of the receiver, while all other RF signals are almost completely suppressed and absent from the output voltage signals, due to the receiver’s frequency selective behavior. The desired RF band of interest is the first harmonic ($n=1$) for which the highest voltage conversion gain is obtained. For the desired RF band signal, the RF signals present at higher harmonics ($n\,\ge \,2$) work as interferers that cannot be completely suppressed by the receiver from its down-converted version of the baseband output signal. In order for the receiver to remain free of interferers and blockers, the receiver is forced to operate only over one octave of the RF frequency range.

When the output is changed from single-ended to differential, the down-converted second harmonic of the RF signal can be cancelled from the output voltage signal; and, the receiver becomes tunable over a much wider RF frequency band. In an ideal situation, the frequency down-converted signal level from the desired RF band increases by 6 dB and the frequency down-converted signal level from the second harmonic gets completely rejected when the output mode is changed from single-ended to differential. Figure 5c provides the measured harmonic rejection of the receiver for 700 MHz band when the output mode was changed from single-ended to differential. All the output signal levels for different harmonics were normalized with respect to the single-ended output mode baseband value for the desired RF band signal (*n* = 1). For the 700 MHz band of operation, about 30 dB suppression for the second harmonic (*n* = 2) of the RF signal from the receiver output was achieved with the differential receiver output mode; and, the desired band (*n* = 1) voltage conversion gain improved by approximately 6 dB.

Ideally, the QPS-FS receiver reflects back all of the blockers outside the passband of the frequency band of operation, with no blockers appearing in the down-converted baseband voltage signal at the receiver output. However, due to hardware impairments and imperfections, some of the blockers do appear at the output of the receiver in real situations, resulting in the reduction of the voltage conversion gain of the receiver for the desired band of operation.

Figure 5d shows the normalized measured receiver voltage conversion gain desensitization due to a CW blocker at a frequency 50 MHz away from the carrier frequency of the band of operation (${f}_{LO}$). In this measurement, the RF and blocker signals were CW signals at frequencies ${f}_{RF}={f}_{LO}$+0.1 MHz and ${f}_{B}={f}_{LO}$+50 MHz, respectively, so that the frequency down-converted baseband signal was obtained at *f*_*IF*_ = 0.1 MHz. For all the tested frequency bands of operation, the receiver could tolerate up to −10 dBm of blocker power when the desired baseband output voltage dropped by approximately 3 dB of its original blocker free level.

**Figure 6:**
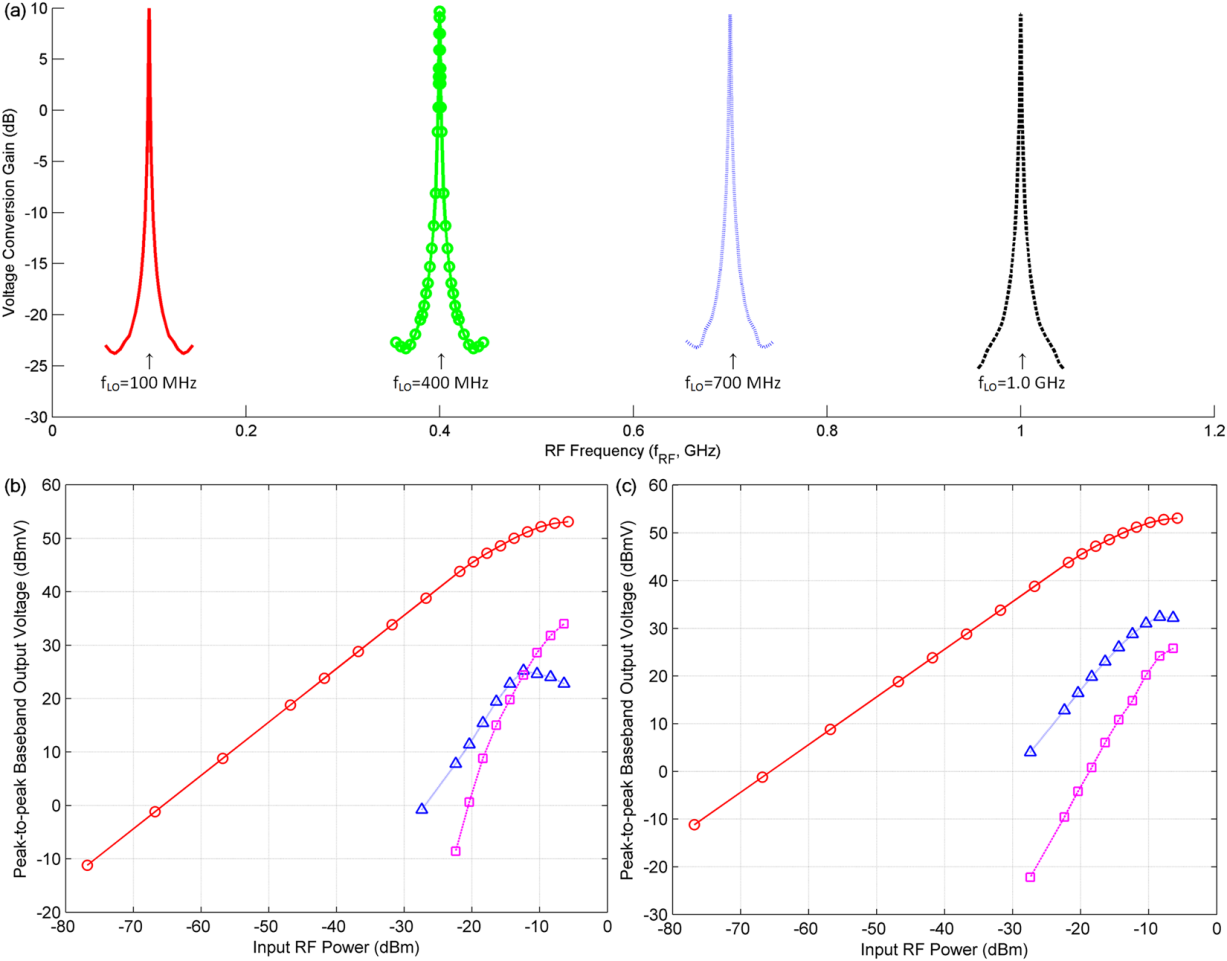
Frequency tunability and nonlinearity characterization of the proposed QPS-FS receiver. (**a**) Frequency tunability of the QPS-FS receiver; receiver voltage conversion gains from RF to baseband frequencies are plotted for LO frequencies of 100 MHz (), 400 MHz (), 700 MHz (), and 1.0 GHz (). **(b)** In-band receiver nonlinearity for 700 MHz band; fundamental (), second-order (), and third-order () in-band receiver nonlinearity characteristics are plotted against the total input RF power. The clock switching frequency is at *f*_*LO*_ = 700 MHz; the baseband output frequency at *f*_*IF*_ = 0.1 MHz; and, the in-band two-tone RF signals are at 701.1 MHz and 701 MHz for the second-order receiver nonlinearity characterizations and at 700.55 MHz and 701 MHz for the third-order. **(c)** Out-of-band receiver nonlinearity for 700 MHz band; fundamental (), second-order (), and third-order () out-of-band receiver nonlinearity characteristics are plotted against the total input RF power; the clock switching frequency is at *f*_*LO*_ = 700 MHz; the baseband output frequency at *f*_*IF*_ = 0.1 MHz; and, the out-of-band two-tone RF signals are at 901.1 MHz and 200 MHz for the second-order receiver nonlinearity measurements and at 550.05 MHz and 400 MHz for the third order. band, in terms of input intercept points ($II{P}_{2}$ and $II{P}_{3}$), for the proposed receiver were 11.6 dBm and 3.5 dBm, respectively.

The receiver is made tunable to any frequency band of operation by changing its local oscillator (LO) frequency equal to the desired band RF signal carrier frequency. The tunability of the receiver is further confirmed experimentally by plotting the voltage conversion gains from RF to baseband frequencies for different LO frequencies shown in Fig. 6a. The receiver is tuned to different RF frequency bands (100 MHz, 400 MHz, 700 MHz and 1.0 GHz RF bands) by only setting the LO frequency (*f*_*LO*_) equal to the desired band RF signal carrier frequency (*f*_*c*_). In this case, the RF signal present at ${f}_{RF}={f}_{c}+{f}_{IF}$ is frequency down-converted to *f*_*IF*_.

When the input RF power was increased, the receiver started to behave nonlinearly, due to the nonlinear behavior of the switches. In order to measure the nonlinearity behavior of the receiver, the peak-to-peak output signal voltages for the baseband signals were recorded in dBmV (=20log_10_(*V*_*pp*_/1 *mV*) and plotted against the total input RF power. In-band and out-of-band receiver nonlinearities were characterized for the 700 MHz RF band of the QPS-FS receiver system. Figure 6a plots the measured output baseband signal levels at frequency *f*_*IF*_ = 0.1 MHz when the two-tone in-band RF signals were sent at frequencies of 701.1 MHz and 701 MHz, so that the down-converted baseband signal due to second order in-band receiver nonlinearity fell at frequency *f*_*IF*_ = 0.1 MHz. Third-order in-band receiver nonlinearity was characterized by two-tone RF signals at frequencies of 700.55 MHz and 701 MHz, so that the down-converted baseband signal due to third-order in-band receiver nonlinearity fell at frequency *f*_*IF*_ = 0.1 MHz. The measured second- and third-order in-band receiver nonlinearity for the 700 MHz band, in terms of input intercept points (IIP2 and IIP3), for the proposed receiver were 11.6 dBm and 3.5 dBm respectively.

The out-of-band receiver nonlinearity for 700 MHz was characterized by two-tone out-of-band RF signals at frequencies of 900.1 MHz and 200 MHz, so that the RF signal due to second-order out-of-band receiver nonlinearity fell at a frequency of 700.1 MHz and the frequency down-converted baseband signal was obtained at *f*_*IF*_ = 0.1 MHz. Similarly, the third-order out-of-band receiver nonlinearity was characterized by two-tone RF signals at frequencies of 550.5 MHz and 400 MHz, so that the RF signal due to third-order out-of-band receiver nonlinearity appeared at a frequency of 700.1 MHz and the frequency down-converted baseband output signal was measured at *f*_*IF*_ = 0.1 MHz. The measured second- and third-order out-of-band receiver nonlinearity characteristics were plotted and are shown in Fig. 6b. The input intercept points for the out-of-band receiver nonlinearity in the 700 MHz band were estimated to be $II{P}_{2}$ = 6.8 dBm and $II{P}_{3}$ = 2.8 dBm.

**Figure 7:**
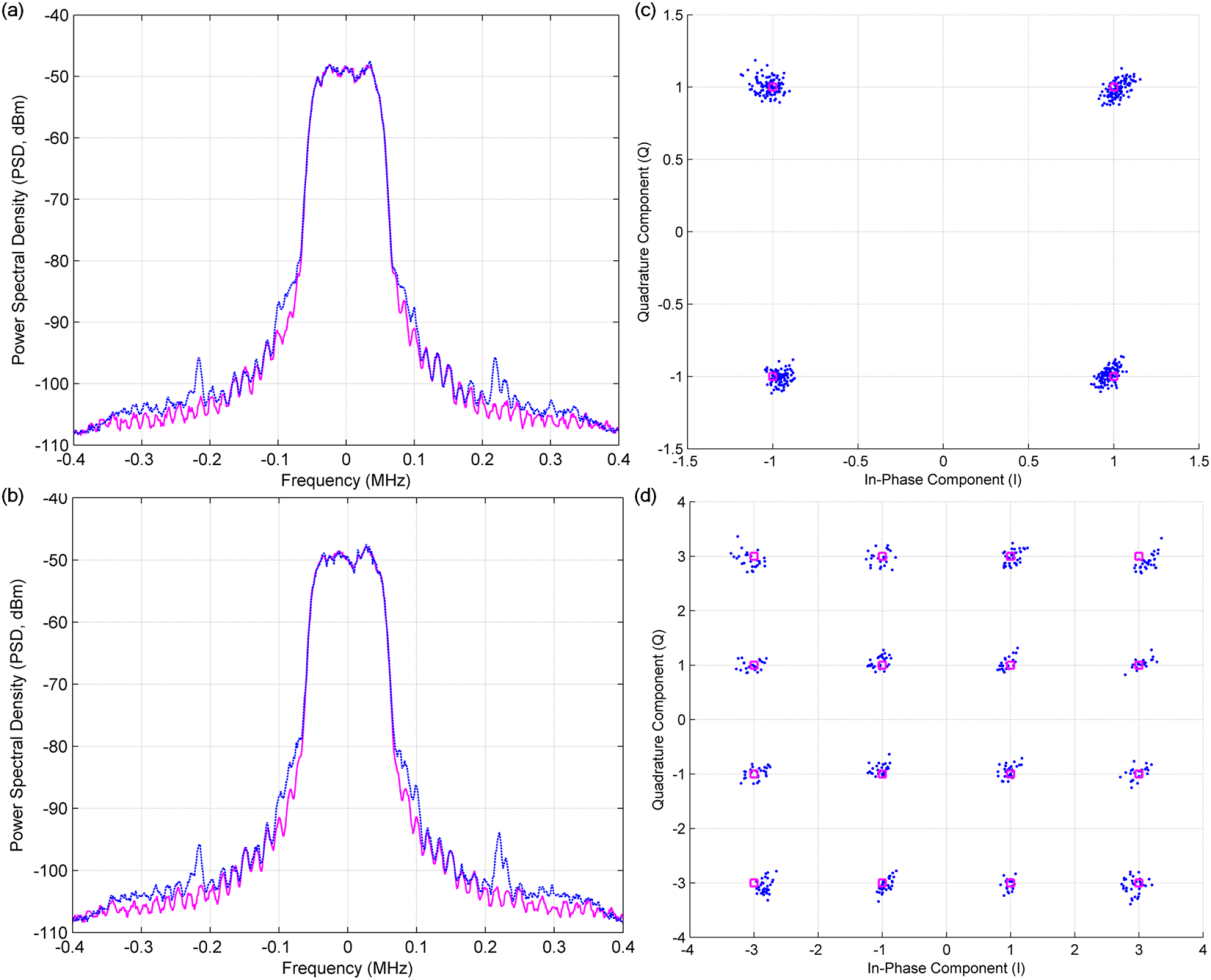
Transmitted and received frequency spectra and constellation points for the proposed QPS-FS receiver. Transmitted () and received () spectra of a **(a)** 4-QAM and **(b)** 16-QAM modulated signal having a 0.1 MHz bandwidth at a 700 MHz carrier frequency. Transmitted () and received () constellation points are also plotted for the (**c**) 4-QAM and (**d**) 16-QAM modulated signal having a 0.1 MHz bandwidth at a 700 MHz carrier frequency.

Figure 7a–d show the transmitted and received spectra and the transmitted and received constellation points, respectively, for 4-QAM and 16-QAM signals having a bandwidth of 0.1 MHz sent and received at a 700 MHz carrier frequency. The modulated RF signal at 700 MHz carrier frequency is obtained from the N5182A vector signal generator. The average approximate RF power for both the signals during measurement was −37 dBm. In this measurement setup, all eight baseband outputs shown in Figs 3b and 4a were directly captured using the sampling oscilloscope working in high impedance mode. The resultant output voltage signals were processed digitally to compensate for any amplitude or phase imbalance in the phase shift network or DC offset from the output signal according to Eq. (7). First 25% of the signal samples were used as training sequences to calibrate for imbalance parameters ${c}_{ij}$ s. The error vector magnitude (EVM) between the transmitted and received constellation points for both the test cases was approximately 4%.

7 $$[\begin{array}{c}I\\ Q\end{array}]=[\begin{array}{cc}\begin{array}{cc}\begin{array}{cc}\begin{array}{c}{c}_{11}\\ {c}_{21}\end{array} & \begin{array}{c}{c}_{12}\\ {c}_{22}\end{array}\end{array} & \begin{array}{c}\cdots \\ \cdots \end{array}\end{array} & \begin{array}{c}{c}_{19}\\ {c}_{29}\end{array}\end{array}]\,{[\begin{array}{ccc}\begin{array}{ccc}1 & {V}_{B1} & {\overline{V}}_{B1}\end{array} & \cdots  & {\overline{V}}_{B4}\end{array}]}^{T}$$

The total power consumption of the proposed receiver is comprised of dynamic power consumed, due to switching of the transistors’ gates and the static power consumed by the differential amplifiers from the DC supplies. There is no additional power needed in generating multiple clocks of reduced speed and duty cycle from a high-speed clock signal using an active multiphase clock generation circuit. The differential amplifiers in the basic test setup were operated on ±2.5 V dual supplies. The dynamic power wasted due to switching of the transistor gates was obtained from simulation in the ADS software using ATF55143 transistor model. All the measured and simulated performance metrics of the complete proposed QPS-FS receiver are summarized in Table 1 for the 700 MHz band of operation.

**Table 1:** Performance summary of the proposed QPS-FS receiver.

Performance metrics	FC	BW	CG	IB IIP_2_	IB IIP_3_	OOB IIP_2_	OOB IIP_3_	HS_2_	CG_D,−10 dBm_	P_D_
**Value**	0.1–1.0	~1.5	9.6	11.6	4.4	6.8	2.8	34.8	3	0.03(−15)
**Unit**	GHz	MHz	dB	dBm	dBm	dBm	dBm	dB	dB	mW(dBm)

**FC** – receiver frequency coverage range.

**BW** – RF down-conversion bandwidth.

**CG** – total combined receiver voltage conversion gain.

**IB IIP**_**2**_ – in-band 2^nd^ order input intercept point.

**IB IIP**_**3**_ – in-band 3^rd^ order input intercept point.

**OOB IIP**_**2**_ – out-of-band 2^nd^ order input intercept point.

**OOB IIP**_**3**_ – out-of-band 3^rd^ order input intercept point.

**HS**_**2**_ – second RF harmonic suppression relative to fundamental harmonic band signal from the receiver output due to change in baseband output mode from single-ended to differential.

**CG**_**D,−10dBm**_ – receiver voltage conversion gain desensitization due to a CW blocker having −10 dBm power present at a frequency 50 MHz away from the desired RF band signal carrier frequency.

**P**_**D**_ – dynamic switching power consumed by the transistor switches obtained from simulation.

Table 2 provides theoretical comparisons of the conventional homodyne, PM, and the proposed QPS-FS receiver architectures.

**Table 2:** Theoretical comparison summary of conventional homodyne receiver (H), passive mixer receiver (P) and the proposed QPS-FS (Q) receiver architectures.

	f_C_	f_LM_	B	J	T	P_S/D_	P	CG	NF	FC
**H**	*f*_*RF*_	*f*_*RF*_	No	No	No	—	High	Low	High	Limited
**P**	*f*_*RF*_	*N* × *f*_*RF*_	Yes	No	Yes	Yes	Moderate	High	Low	Limited
**Q**	*f*_*RF*_	*f*_*RF*_	Yes	Yes	Yes	No	Low	Moderate	Moderate	Wider

**f**_**C**_ – RF signal carrier frequency.

**f**_**LM**_ – master LO clock source frequency.

**B** – is the receiver blocker tolerant.

**J** – is the receiver clock jitter tolerant.

**T** – is the receiver frequency selective and tunable.

**P**_**S/D**_ – static and dynamic power consumed by the multi-phase clock generation circuit/transistors.

**P** – total static/dynamic power consumed by the receiver.

**CG** – receiver voltage conversion gain.

**NF** – receiver noise figure.

**FC** – frequency coverage of the receiver given a master LO clock source of fixed maximum frequency.

In summary, the conventional passive mixer receivers are known in the art to have high linearity (due to passive circuit element involvement, i.e. switch) and high selectivity (due to impedance translation property) compared to conventional homodyne architectures but lack performance in terms of gain and noise figure^19^. Indeed, the proposed QPS-FS receiver utilizes two transistors impedance translation circuit in each of the four paths of the phase shift network comprising of passive circuit elements (hybrid circuits). Due to this, the QPS-FS receiver suffers in terms of gain and noise figure but its linearity and selectivity is superior to the conventional homodyne architectures. These well-known architectural features of the conventional homodyne receiver architectures and the passive mixer architectures in comparison to the proposed QPS-FS receiver architecture have been summarized in Table 2. The conventional passive mixer receivers employ an additional multi-phase clock generator circuit that converts a single high frequency clock signal into multiple same speed clock signals with reduced duty cycles. This multi-phase clock generator circuit consumes additional power in the conventional passive mixer architecture. The need for high frequency clock signal and additional power consuming multi-phase clock generator circuit have been eliminated from the QPS-FS receiver thus reducing the overall power consumption of the QPS-FS receiver and extending its frequency coverage in comparison to the conventional passive mixer receivers. In addition to that, the QPS-FS receiver is tolerant to clock jitter as confirmed through simulation.

Table 3 provides performance comparison of some recent reported results of homodyne receivers, passive mixer receivers, and the proposed QPS-FS receiver.

**Table 3:** Performance comparison of some recent reported conventional homodyne receivers, passive mixer receivers and the proposed QPS-FS receiver architectures.

Ref.	Architecture Core	Mixer Type	RF Input	Frequency (GHz)	Conversion Gain (dB)	IIP_2_ (dBm)	IIP_3_ (dBm)	Technology
35	Noise and Linearity performance enhanced; Modified Gilbert cell topology	Active	Differential	0.5–5.8	16.3–14.4	—	7.3–2.5	0.13 um CMOS
36	Linearity enhanced; Modified Gilbert cell topology	Active	Differential	0.5–6.5	11.2–6.9	—	9.52	0.13 um CMOS
37	Linearity enhanced; Subharmonic mixer topology	Active	Differential	2.4	8.5	88	−0.1	0.18 um CMOS
20	Improved harmonic rejection; 8-path passive mixer	Passive	Differential	0.5–2.5	~35	>50	14	28 nm CMOS
29	LO leakage suppression; 8-path passive mixer	Passive	Single-ended	0.4–3.5	35	>60	16	28 nm CMOS
**This work**	4-path QPS-FS	Passive	Single-ended	0.1–1.0	9.4	11.6	6.8	PCB

## Discussion

In the ideal proposed QPS-FS receiver, all of the blocker and interferer power outside the passband of the receiver band of operation is reflected, with no appearance in the output baseband voltages taken from the output capacitors. Half of the blockers’ power is reflected back to the 180° hybrid, while the other half is dissipated in the matched isolation resistor terminations (*T*_1_ and *T*_2_ shown in Fig. 3b). Each of these terminations absorbs blocker/interferer power that is 6 dB less than the total input blocker/interferer power in the receiver. For a CW blocker presented at 750 MHz having −10 dBm power at the receiver input, along with a desired RF signal at 700.1 MHz $({f}_{RF})$ having −33 dBm power, the actual measured blocker powers in *T*_1_ and *T*_2_ were −17.6 dBm and −18.2 dBm, respectively, at 750 MHz, which were 1.6 dB and 2.2 dB less than the interferer power levels that would have been obtained with an ideal receiver. This measurement confirmed the blocker tolerant behavior of the QPS-FS receiver system. Measured reflected blocker power that is less than the ideal value can be attributed to loss in the phase shift network, impedance mismatches and the finite (nonzero) stopband impedance of the ITCs.

Although it is outside the scope of this article to implement an actual energy harvesting system, the blocker/interferer power reaching hybrid port terminations can be combined and supplied to an actual wideband RF-to-DC rectifier as an energy harvesting system, where the RF power is converted to DC power and stored for further usage.

## Conclusions

A novel radio frequency (RF) blocker and local oscillator (LO) clock jitter tolerant receiver architecture has been proposed in this article. The receiver architecture is linear and it uses passive signal division networks in the RF and the LO paths of the receiver. The proposed quadrature phase shift frequency selective (QPS-FS) receiver is passive, frequency selective and tunable compared to conventional homodyne architectures, while requiring a much slower clock signal source than passive mixer (PM) based receivers, thus significantly reducing the needed power consumption of the proposed receiver architecture and extending the frequency coverage of the receiver to the maximum of the source clock frequency. The proposed QPS-FS receiver employs a linear and passive phase shift network in the RF path and slower speed complementary clock signals that can be directly connected to the switching transistors’ gates. Elimination of an active multiphase clock generation circuit and reduction of the operating frequency decreases the overall power consumption of the proposed receiver system. Sharing of common clock signals by the switching transistors helps in reducing the effect of clock jitters on the overall receiver performance. The performance of an actual implemented receiver system using the proposed QPS-FS receiver architecture was verified for the 700 MHz band of operation. The undesired interferer signals could be isolated from the desired RF signal and collected in the proposed receiver for energy recycling purpose.
